# Machine Learning-Based Pitting Rate Classification and Prediction for 316L Stainless Steel in NaClO_3_ and NaCl Environment

**DOI:** 10.3390/ma19101979

**Published:** 2026-05-11

**Authors:** Cheng Zhang, Jiaxin Yao, Zhe Zhang

**Affiliations:** 1 School of Chemical Engineering and Technology, Tianjin University, Tianjin 300350, China; frank_z@tju.edu.cn (C.Z.); 2023207002@tju.edu.cn (J.Y.); 2 Zhejiang Institute of Tianjin University, Ningbo 315201, China

**Keywords:** machine learning, ADASYN, FFNN, pitting rate classification, 316L stainless steel, NaClO_3_/NaCl solution

## Abstract

The 316L stainless steel is widely utilized as structural material in hydrogen production industry due to its excellent combination of corrosion resistance and mechanical properties. However, it remains susceptible to localized pitting corrosion in chloride-containing high-temperature environments. Especially, the main electrolysis byproduct sodium chlorate (NaClO_3_) also has complicated effect on pitting corrosion. Therefore, evaluating and predicting the pitting severity grades of 316L steel in NaClO_3_ and NaCl environment is essential for controlling operation risks. In recent years, machine learning (ML) methods have gained significant attention in the field of corrosion prediction; however, existing research has primarily focused on the regression prediction of continuous parameters, while studies dedicated to the classification and evaluation of pitting severity grades remain relatively limited. Furthermore, experimental datasets are commonly constrained by small sample sizes and imbalanced class distributions, which hinder the performance enhancement of classification models. Based on experimental pitting data of 316L stainless steel, this study employs ADASYN (Adaptive Synthetic Sampling) to mitigate data imbalance and develops a Feedforward Neural Network (FFNN) for pitting rate classification. The proposed model is compared and analyzed against several commonly used machine learning models. Through a comprehensive evaluation of predictive performance, the feasibility of the developed model in pitting severity grading is verified, thereby providing a novel approach for the predictive evaluation of the pitting corrosion of 316L stainless steel.

## 1. Introduction

Hydrogen production by electrolysis of sodium chloride (NaCl) is one of important methods for hydrogen industry. As an indispensable structural material, the reliability of 316L stainless steel directly determines the safety and lifespan of engineering infrastructure. However, the chromium oxide passive film on the surface of 316L stainless steel is highly susceptible to damage in chloride-containing environments, which subsequently triggers localized pitting corrosion [1,2,3,4]. In practical electrolysis of sodium chloride process, sodium chlorate (NaClO_3_) is the main electrolysis byproduct. It has been reported that the NaClO_3_ significantly deteriorates corrosion resistance due to its potent oxidizing properties [5,6,7]. However, it is also reported that a competitive adsorption exists between the NaCl and NaClO_3_, which tends to restrict the pitting behavior [8]. Although existing studies have acknowledged the presence of ClO_3_^−^, its mechanistic role in complex multi-ionic environments remains complicated [5,6,7,8,9]. Consequently, it is of critical importance to elucidate the pitting behavior of 316L stainless steel under the synergistic effect of NaCl and NaClO_3_ solution, and to establish accurate methodologies for pitting prediction and assessment.

Traditional pitting corrosion evaluation for stainless steel primarily relies on a series of methods outlined in ASTM/ISO standards, including cyclic polarization testing (ASTM G61 [10]), critical pitting temperature (CPT) measurement (ASTM G150 [11]), ferric chloride pitting tests (ASTM G48 [12]), and pitting evaluation based on surface morphology (ASTM G46 [13]). These methods evaluate material pitting susceptibility and service risks through indicators such as electrochemical parameters, pit depth and density, and maximum pit size. However, these approaches generally depend on identification and evaluation of corrosion that has already occurred, leading to significant time-lag, passive response, and limited predictive capability. In recent years, Machine learning (ML) has demonstrated new research potential in the field of pitting evaluation and prediction. Existing studies have shown that ensemble models based on Support Vector Machines (SVMs) exhibit favorable performance in predicting pitting corrosion of stainless steel, such as the research by Jiménez-Come et al. on the pitting behavior of EN 1.4404 stainless steel [14,15,16]. With the rapid development of artificial intelligence, Artificial Neural Networks (ANNs) have also been widely applied in corrosion prediction. For instance, Jafari et al. utilized ANN to predict the corrosion behavior of AISI 316L stainless steel under various surface treatment conditions [17]. Moreover, Aghaaminiha et al. employed several supervised learning methods to predict the corrosion rate of carbon steel in inhibitor-containing environments [18]. Furthermore, Jiménez–Come et al. developed an automated method using ML techniques, including ANN, to predict the occurrence of pitting in 316L stainless steel under diverse environmental conditions [19]. Feedforward Neural Networks represent a class of fully connected neural structures comprising an input layer, hidden layers, and an output layer, where information propagates unidirectionally through the network. Compared to models designed for specific data structures, such as Convolutional Neural Networks or Recurrent Neural Networks, FFNNs have lower requirements for input data formats and are suitable for modeling tasks with limited sample sizes and relatively well-defined feature dimensions. With their robust nonlinear mapping capabilities and adaptive learning features, FFNNs demonstrate significant potential in predicting material corrosion behavior [20,21,22].

Despite the immense potential of machine learning in corrosion science, its application to pitting assessment still faces many challenges. On the data level, constrained by long experimental period and high cost, pitting datasets are typically characterized by limited sample sizes and imbalanced class distributions. These factors often lead to model overfitting, thereby hindering the generalizability and practical implementation of ML algorithms. On the application level, existing research has predominantly focused on continuous quantitative regression of corrosion parameters, which often falls short of meeting the industrial demand for discrete categorical grading of pitting severity. Consequently, developing a high-reliability predictive model for pitting severity grading under conditions of small-scale, imbalanced data has become a critical focus in current machine learning-based corrosion damage assessment. To mitigate the decision bias caused by data imbalance, oversampling techniques are frequently employed to artificially expand the minority class and reshape the data distribution [23,24,25,26]. Similarly, in the field of corrosion, numerous studies have employed oversampling techniques to balance and augment datasets, thereby optimizing feature extraction and enhancing the sensitivity of decision boundaries [27,28,29]. However, in practical applications, traditional Random Oversampling is highly susceptible to triggering overfitting. In addition, the classic SMOTE (Synthetic Minority Over-sampling Technique) algorithm does not account for the varying learning difficulties of different samples when generating new data, which can lead to the blurring of inter-class decision boundaries. In contrast, Adaptive Synthetic Sampling achieves data balance by generating synthetic minority class samples while incorporating an adaptive mechanism that prioritizes hard-to-classify samples [30]. Therefore, the ADASYN has great potential for improve the ML predicted accuracy. Ahmed et al. utilized ADASYN to propose an improved model for Alzheimer’s disease classification [31]. Saqib et al. employed an ADASYN-enhanced model to improve electricity theft detection [32]. Additionally, Zhang et al. introduced an intelligent rockburst prediction model based on an optimized LightGBM framework, integrating ADASYN with the Whale Optimization Algorithm (WOA) [33]. However, the application of ADASYN in corrosion damage assessment is still inadequate.

Based on the aforementioned considerations, in this study, an ADASYN-FFNN pitting rate classification model is constructed to be trained and tested on experimental pitting data of 316L stainless steel in NaCl and NaClO_3_ cooperative environments. By integrating ADASYN with a specifically designed FFNN structure, this research proposes to mitigate overfitting under small-sample conditions, thereby enhancing the model’s generalization capability and predictive accuracy. Furthermore, a comparative analysis with baseline models, including Random Forest (RF), Support Vector Machine (SVM), and K-Nearest Neighbors (KNN), is conducted to validate the superiority and applicability of the proposed model in predicting the corrosion damage of 316L stainless steel.

## 2. Methodology

### 2.1. Immersion Test Methods

**Figure 1:**
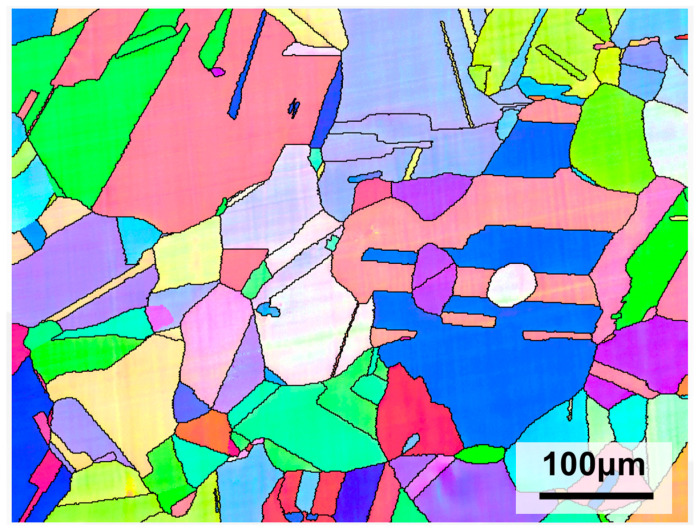
Microstructure of 316L stainless steel.

In this study, commercial 316L stainless steel plates were employed, and the chemical composition is listed in Table 1 [8]. As illustrated in Figure 1, the as-received 316L steel exhibits a homogeneous equiaxed grain structure with an average grain size of 90 μm.

The specimens were prepared as perforated coupons with dimensions of 20 mm × 10 mm × 1 mm. Prior to testing, the specimens were sequentially ground using 180, 400, 1000, and 2000-grit SiC papers. Subsequently, mechanical polishing was performed on the surface using 1 μm diamond paste. After eliminating surface scratches, the specimens were rinsed with deionized water and ethanol, followed by air-drying for later use.

Based on the actual structural conditions of the NaCl electrolysis section, the corrosive medium used in the experiments was a mixed NaCl/NaClO_3_ solution, prepared with a saturated NaCl solution as the base and additions of 6, 20 and 40 g/L NaClO_3_. The pH of the solution was adjusted to 2 using diluted HCl. For immersion tests conducted at 90, 120 and 150 °C, PTFE-lined steel autoclaves were utilized as containers, with heating provided by a constant-temperature drying oven. The 200 °C immersion tests were carried out using an integrated hydrothermal reactor. The total duration of the immersion experiment was 30 days, with sampling intervals at 7, 14, 21 and 30 days. At each time point, specimens were retrieved for surface observation and analysis. If the specimen surfaces were heavily covered with corrosion products, they were cleaned via ultrasonic treatment in an oxalic acid solution to enable the continuous observation and measurement of corrosion pits on the same specimen. Following the analysis, the solution was refreshed to compensate for the consumption of effective corrosive species and maintain the target concentration of the corrosive environment.

**Figure 2:**
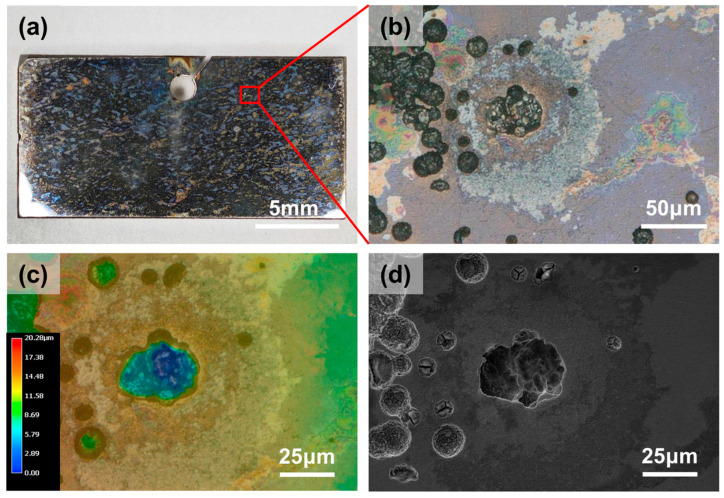
Surface morphologies of 316L stainless steel after immersion at 120 °C: (**a**) Digital camera, (**b**) Optical microscope (OM), (**c**) OM 3D, and (**d**) SEM.

Microscopic characterization was performed using a Keyence VHX-5000 digital microscope (Keyence (China) Co., Ltd., Shanghai, China) and a Phenom XL Scanning Electron Microscope (FEI Electron Optics BV, Eindhoven, The Netherlands) to observe and measure the corroded specimen surfaces, as shown in Figure 2. Subsequently, the maximum pitting depth was recorded, and the pitting rate was calculated. The pitting rates were then categorized according to the SY/T 0087.1 standard [34]. A total of 53 sets of experimental data were statistically compiled. The classification standards for pitting rates and partial experimental results are presented in Table 2 and Table 3.

### 2.2. Development of ADASYN-FFNN Model

In pitting assessment research, relevant experimental data primarily originate from electrochemical parameters or statistical features. The data structure of these parameters typically differs from image or sequential data, which possess explicit spatial or temporal correlations. Against this background, the FFNN as a general-purpose neural network architecture, can establish the relationship between the input features and pitting severity grades through multi-layer nonlinear mapping. Simultaneously, it maintains a relatively concise model structure, facilitating subsequent performance optimization in combination with regularization or data processing methods. Existing studies have demonstrated that FFNNs exhibit favorable stability and applicability in corrosion prediction and related tasks [35,36,37,38]. Based on these considerations, in this study, the PyTorch (v2.2.2) framework was employed to construct an FFNN-based pitting rate classification model to accurately evaluate and predict the corrosion severity of 316L stainless steel.

**Figure 3:**
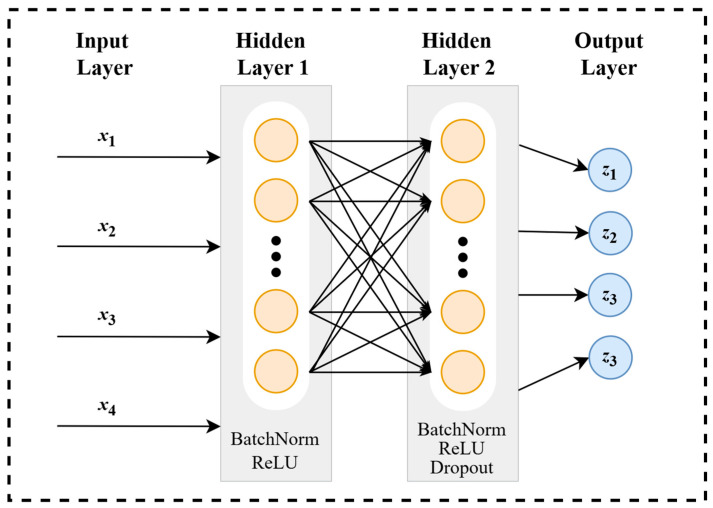
Architecture of the FFNN model.

The overall model architecture is illustrated in Figure 3. The input variables, *x*_1_*, x*_2_*, x*_3_, and *x*_4_, represent the temperature, NaClO_3_ concentration, pH value, and immersion time of the immersion experiments, respectively. The output results correspond to the four grades defined in the pitting rate classification standards, where Grades I to IV are encoded as numerical labels 0 to 3, respectively. For the data obtained from the aforementioned immersion experiments, the ADASYN oversampling method is employed to enrich the samples, thereby avoiding the overfitting problem triggered by a small sample size. Meanwhile, to evaluate the impact of the oversampling strategy on model performance, a comparative analysis is conducted against an identical FFNN model trained without any data resampling in this study.

Regarding the number of hidden layers, it is theoretically postulated by the Universal Approximation Theorem that a feedforward neural network with at least one hidden layer can approximate any continuous function to any desired degree of accuracy [39,40,41]. However, in practical applications, it has been demonstrated that the implementation of merely a single hidden layer does not inherently guarantee predictive accuracy equivalent to that of deeper networks. As highlighted in the recent literature [42], architectures incorporating two or more hidden layers are frequently required to improve the repeatability of the learning process and achieve superior accuracy. Therefore, considering the computational training costs and the sample size, in this study, a two-hidden-layer structure and the ReLU (Rectified Linear Unit) activation function were adopted, while simultaneously incorporating BatchNorm and Dropout layers to prevent the model overfitting. The BatchNorm layer stabilizes the inputs by performing batch normalization on the outputs of the hidden layers, thereby enhancing the model’s generalization capability and accelerating the optimization of model fitting [43]. Moreover, the Dropout layer randomly deactivates a fraction of neurons during the training process, which suppresses feature co-adaptation and achieves random sparsification of the network structure, ultimately improving the generalization capability of the model and reducing the risk of overfitting [44].

### 2.3. Model Training

**Figure 4:**
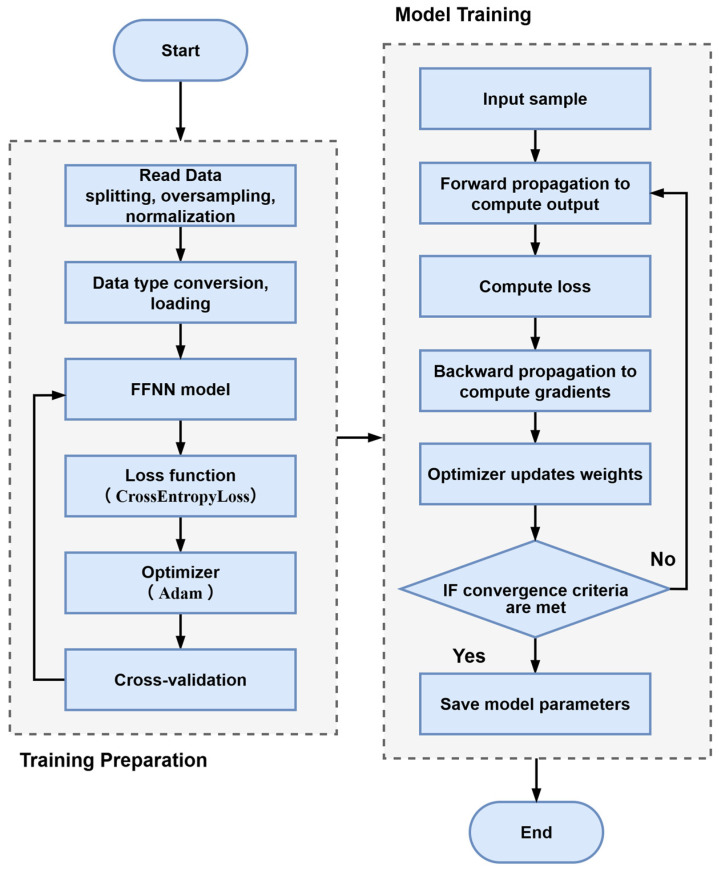
Flowchart of ADASYN-FFNN training.

The overall model training process is illustrated in Figure 4. First, the experimental data must be partitioned into a training set and a testing set. Subsequently, the ADASYN oversampling technique is applied to the training data to achieve an enriched and balanced training set. Following this, data normalization is performed on both sets to mitigate the impact of data magnitude variations on the training process, utilizing the MinMaxScaler from the scikit-learn library. The data types are then converted to complete the definition of the Dataset and the loading of the DataLoader. CrossEntropyLoss is selected as the loss function, and Adam is chosen as the optimizer. Primarily, this loss function is highly suitable for multi-class classification tasks, demonstrating faster convergence and superior numerical stability during training [45]. Furthermore, as one of the most widely used optimization algorithms in deep learning, the Adam optimizer combines the principles of Momentum and RMSProp (Root Mean Square Propagation). It generally achieves faster and more stable convergence when training neural networks compared to traditional SGD (Stochastic Gradient Descent) [46].

Although the overall architecture of the FFNN model has been introduced above, the specific number of neurons in the two hidden layers remains undetermined. Generally, parameters that govern the architecture of a machine learning model are referred to as hyperparameters [47]. The cross-validation is an effective method for determining optimal hyperparameters. Its core concept involves partitioning the samples into multiple folds, followed by iteratively training the model. In each iteration, a different subset is selected as the validation set, while the remaining subsets are used for training. This allows for multiple evaluations of the model’s performance, the results of which are ultimately averaged. A widely utilized approach is K-Fold Cross-Validation, where the dataset is divided equally into K partitions. Initially, the first fold serves as the validation set while the remaining K-1 folds are used for training. This process is repeated sequentially until the K-th fold, yielding K evaluation metrics that are subsequently averaged. In this study, 5-fold cross-validation is selected. The number of neurons for each of the two hidden layers is varied from 5 to 20, creating various combinations, and cross-validation is performed on all possible combinations. The subsequent training steps proceed according to the workflow depicted in the figure, concluding with the preservation of the final trained model.

During the model training phase, the inherent stochasticity of the neural network’s learning process was explicitly taken into account. It is recognized that the weight initialization and gradient descent pathways can lead to fluctuations in performance, even when the same training set is utilized. To mitigate this effect and ensure the reliability of the results, each architectural configuration was subjected to multiple independent training runs. As emphasized in recent research [42], this approach is essential to evaluate the repeatability of the learning process and to identify configurations that demonstrate both high accuracy and high stability.

### 2.4. Other Machine Learning Algorithms

In parallel with the FFNN, several other machine learning algorithms, namely SVM, Random Forest, and KNN, were also employed. For these three algorithms, a cross-validation process was similarly conducted prior to training to ensure that the relevant hyperparameters for each algorithm reached optimal values.

The primary concept of SVM is to identify a hyperplane that separates different data classes, while maximizing the margin (distance) between the data points of the two classes and the hyperplane. For the SVM model, the SVC class from the sklearn.svm module was utilized. The hyperparameters subjected to cross-validation included the regularization parameter (*C*) and the gamma parameter (*γ*) of the Radial Basis Function (RBF) kernel. The hyperparameter search during the cross-validation was executed using Bayesian optimization via the Tree-structured Parzen Estimator (TPE) algorithm within the Optuna framework. Compared to traditional exhaustive grid search methods, Optuna effectively reduces invalid computations, thereby decreasing computational time [48].

Random Forest is an algorithm within the domain of Ensemble Learning. Its core philosophy involves constructing a classifier comprising multiple decision trees, where the final classification result is determined by the mode of the classes outputted by individual trees. For the Random Forest model, the RandomForestClassifier from sklearn.ensemble was adopted. Regarding its hyperparameter tuning, four common hyperparameters were selected: the number of trees in the forest (n_estimators), the minimum number of samples required to be at a leaf node (min_samples_leaf), the maximum depth of the tree (max_depth), and the minimum number of samples required to split an internal node (min_samples_split). The cross-validation was similarly conducted using Optuna (v4.5.0).

KNN is a simple yet widely used classification algorithm. It operates by calculating the distances between the sample to be classified and the samples in the training set to identify the *K* nearest neighbors, and then classifies the sample based on the majority class among these *K* neighbors. For the KNN model, the KNeighborsClassifier from sklearn.neighbors was employed. The hyperparameters selected for cross-validation included the number of neighbors (n_neighbors), the weight function used in prediction (weights), and the distance metric (metric), with the Optuna framework again utilized for optimization.

## 3. Results and Discussion

### 3.1. Immersion Corrosion Test Results

**Figure 5:**
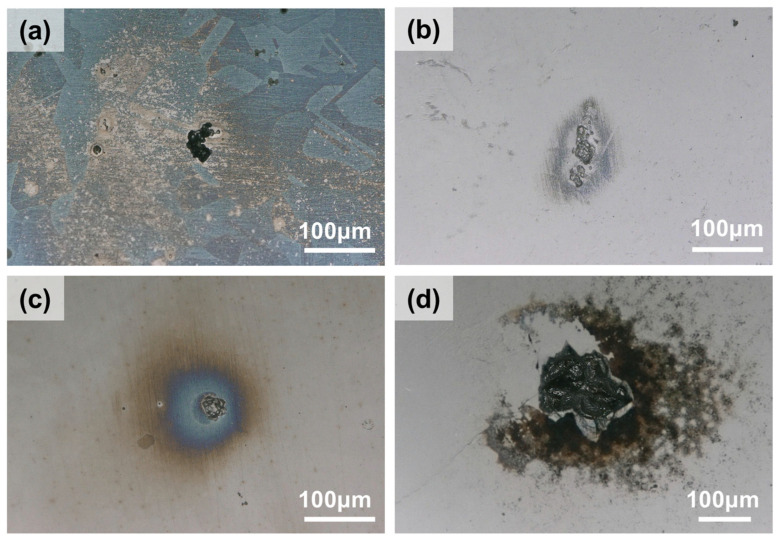
Surface morphologies of 316L stainless steel after immersion in saturated NaCl solution at 120 °C for 7 days with: (**a**) no NaClO_3_, (**b**) 6 g/L NaClO_3_, (**c**) 20 g/L NaClO_3_, and (**d**) 40 g/L NaClO_3_.

Figure 5 displays the corrosion morphologies of 316L stainless steel immersed at 120 °C with varying concentrations of NaClO_3_. As the NaClO_3_ concentration increases, the maximum pitting depth exhibits a trend of initially decreasing and subsequently increasing. On the sample without NaClO_3_ addition, several micro-pits are scattered around the primary pitting hole. With the addition of 6 g/L NaClO_3_, the sample surface remained relatively clean, and the shallowest maximum pitting depth of 5 μm was recorded. At a concentration of 20 g/L NaClO_3_, the maximum pit depth was found to be 18 μm, although the pit area showed little variation compared to the lower concentration groups. Ultimately, the most severe localized corrosion is observed in the NaCl solution with 40 g/L NaClO_3_, wherein the maximum pitting depth reaches 60 μm, accompanied by the large pit area.

**Figure 6:**
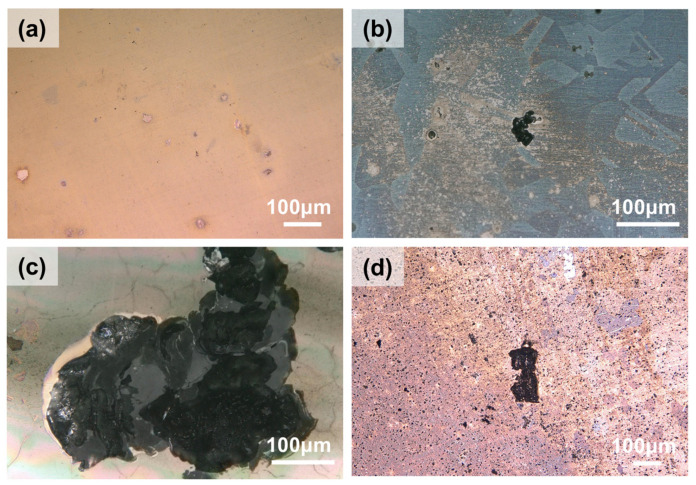
Surface morphologies of 316L stainless steel after immersion in saturated NaCl solution for 7 days: (**a**) at 90 °C, (**b**) at 120 °C, (**c**) at 150 °C, and (**d**) at 200 °C.

**Figure 7:**
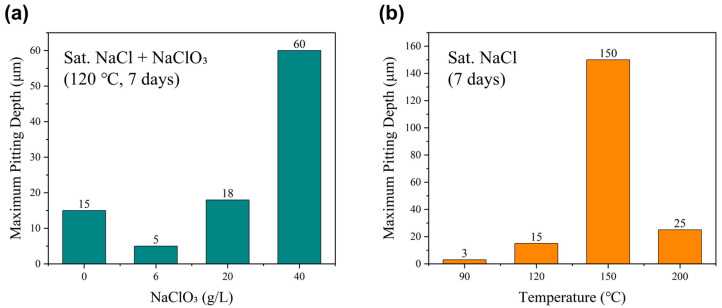
Maximum pitting depth after immersion for 7 days in the saturated NaCl solution: (**a**) NaClO_3_ concentration effect; (**b**) immersion temperature effect.

Figure 6 illustrates the surface morphologies of the samples after immersion for the same duration at different temperatures. Evidently, at 90 °C, the degree of pitting is relatively mild, characterized by small pit areas and shallow depths. At 120 °C, larger pits appear on the surface with a depth of approximately 15 μm. As the immersion temperature further increases, the severity of pitting escalates drastically; at 150 °C, the maximum pit depth reaches 150 μm, accompanied by a substantial expansion in pit area. Interestingly, when the temperature is elevated to 200 °C, numerous fine micro-pits emerge on the surface due to the uniform corrosion of the surface, and the maximum depth of the larger pits is only 25 μm. A comparison of the maximum pitting depths is provided in Figure 7. In summary, the most severe pitting is observed at the intermediate temperature of 150 °C, while the maximum pitting depth is found to be significantly reduced at immersion temperature of 200 °C due to the occurrence of extensive uniform corrosion.

**Figure 8:**
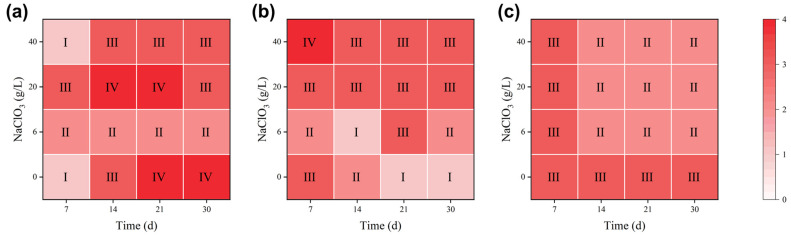
Heatmaps of pitting rate grades for 316L stainless steel: (**a**) at 90 °C, (**b**) at 120 °C, and (**c**) at 150 °C.

The heatmaps in Figure 8 indicate that the evolution of pitting rate grades with immersion time and NaClO_3_ concentration displays highly discrete and complex nonlinear features. It is evident that the evolutionary trends over time at identical concentrations differ significantly across temperatures (e.g., an unexpected decrease in pitting grade over time at 120 °C with 0 g/L NaClO_3_). Furthermore, the high-temperature system (150 °C) fails to reach the severe Grade IV. Such complexity in the pitting behavior of 316L stainless steel, induced by multiple variables including temperature, time and oxidant concentration, makes it difficult for traditional empirical formulas to accurately capture the intrinsic rules. This effectively demonstrates the fundamental advantage of utilizing machine learning in this research: relying on its powerful data-driven capacity to precisely extract the nonlinear mapping relationships concealed within high-dimensional data, thus achieving more reliable predictions for corrosion evolution.

### 3.2. Optimal Hyperparameters of the Models

The cross-validation hyperparameters for the four algorithms were introduced in the preceding sections. The selection of their specific search ranges was based on references from existing literature [18,49,50,51]. The detailed hyperparameter ranges are presented in Table 4.

It is crucial to select an appropriate evaluation metric for determining the optimal hyperparameters. Accuracy (ACC) is undoubtedly a highly effective evaluation metric; it is straightforward, intuitive, and comprehensively reflects the model’s performance under various parameter configurations. Accuracy is defined as the proportion of correctly classified samples relative to the total number of samples. Let *N_correct_* denote the number of correctly predicted samples (i.e., instances where the predicted class matches the true class), and *N_total_* denote the total number of samples. Then, the accuracy can be calculated as follows:

(1) $$\mathrm{ACC}=\frac{N_{correct}}{N_{total}}$$

A higher accuracy value indicates a superior predictive classification capability of the model.

**Figure 9:**
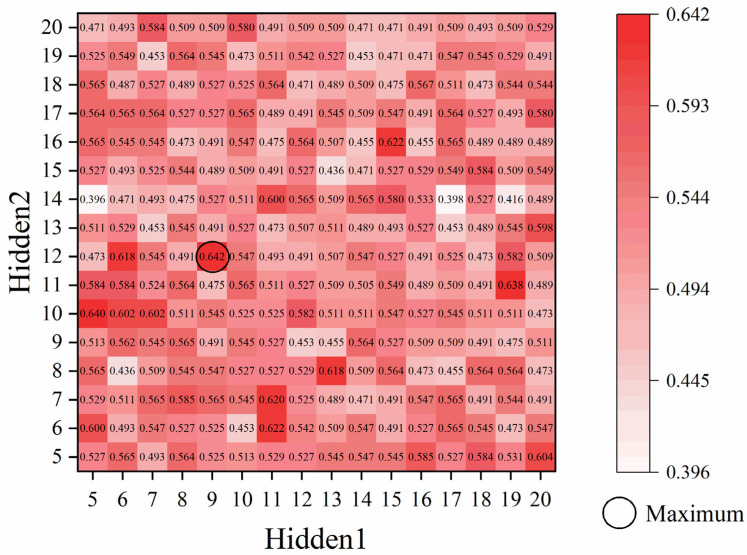
Heatmap of cross-validation accuracy for the FFNN model.

Upon completion of the cross-validation procedure, the ACC values for various hyperparameter combinations are obtained, and the specific hyperparameters corresponding to the maximum ACC are selected through comparison. Taking the FFNN as an example, a heatmap depicting the ACC with respect to the two hidden layers is generated. As illustrated in Figure 9, the results indicate that the optimal ACC is achieved when the number of neurons is 9 in the first hidden layer (Hidden1 = 9) and 12 in the second hidden layer (Hidden2 = 12). As established in the training methodology, the optimal 9/12 configuration was selected, not only for its peak performance but for its superior stability across multiple stochastic repetitions, as well. After conducting similar hyperparameter selection procedures during the cross-validation of the other algorithms, the optimal hyperparameters for all models are determined and summarized. Subsequently, model training and testing are executed based on the parameters established in Table 5.

It is worth mentioning that a total of 53 original samples were utilized for the entire process, with 80% (42 samples) and 20% (11 samples) assigned to the training and test sets, respectively. To mitigate the effects of data imbalance, the ADASYN algorithm was applied exclusively to the training set, resulting in an expanded training size of 91 samples. The test set was kept in its original state to provide a realistic evaluation of the predictive accuracy.

### 3.3. Predictive Performance of the Models

#### 3.3.1. Evaluation Metrics

During the testing phase, to evaluate the model’s performance more deeply and comprehensively, Precision (PRE) and Recall (REC) were further introduced as performance evaluation metrics. Precision denotes the proportion of samples predicted as a specific class that genuinely belong to that class. Recall represents the proportion of samples actually belonging to a specific class that are correctly identified by the model. For a given class, let *TP* (True Positive) be the number of instances correctly predicted as this class, *FP* (False Positive) be the number of instances incorrectly predicted as this class, and *FN* (False Negative) be the number of instances actually belonging to this class but incorrectly predicted as another class. Then, the PRE and REC can be calculated as follows:

(2) $$\mathrm{PRE}=\frac{TP}{TP+FP},$$

(3) $$\mathrm{REC}=\frac{TP}{TP+FN}.$$

Since the individual PRE and REC values for each class cannot directly reflect the overall performance of the model, their weighted averages, calculated based on the number of samples in each class, are typically utilized for a comprehensive evaluation.

#### 3.3.2. Performance Score Comparison

**Figure 10:**
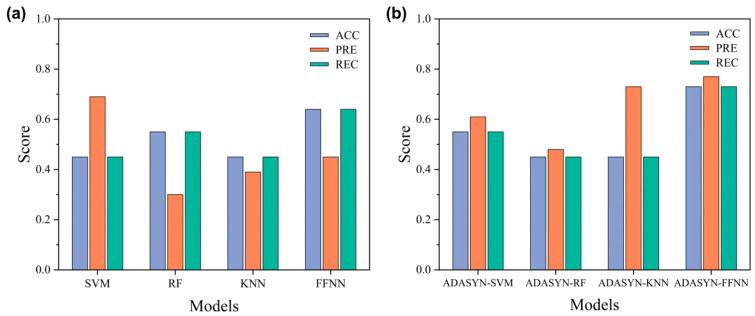
Comparison of performance scores for different models: (**a**) trained on the original dataset; (**b**) trained on the dataset after ADASYN oversampling.

Figure 10 presents a detailed comparison of the ACC, PRE, and REC of the four models (SVM, RF, KNN, and FFNN) on the original dataset and after ADASYN oversampling. On the original dataset without oversampling, the performance of the models exhibits significant divergence. Among the traditional machine learning models, the SVM demonstrates a relatively high PRE (0.69), but its ACC and REC are merely 0.45. This indicates that its decision boundary leans towards conservatism; while it controls false alarms, it severely sacrifices the recognition rate of the majority class samples. RF achieves a REC of 0.55, but a low PRE of 0.30, suggesting that in its attempt to improve recall, it introduces a large number of misclassified samples, thereby limiting its discriminant precision. The KNN shows no advantage across any metrics, highlighting its high sensitivity to the class imbalance problem. In contrast, the FFNN demonstrates a significant baseline advantage, with both ACC and REC reaching 0.64. It not only comprehensively outperforms the traditional models but preliminarily validates its superior capability in fitting complex, nonlinear pitting corrosion grades as well.

Following the introduction of ADASYN oversampling, the models exhibit marked differences in their capacity to assimilate the synthetic samples. The improvements in traditional models are highly limited and even show performance deterioration: although the ACC of SVM increases to 0.55, it comes at the expense of a partial loss in PRE. The overall performance of RF declines rather than improves, exposing the extreme sensitivity of tree-based ensemble algorithms to the local noise introduced by synthetic samples. Despite KNN jumping to a PRE of 0.73, its core metrics, ACC and REC, remain stagnant. In stark contrast, the ADASYN-FFNN model achieves a comprehensive breakthrough across all three metrics—its ACC and REC increase to 0.73, and its PRE reaches an impressive 0.77. Compared to the baseline FFNN, this combined model significantly reduces the false alarm rate while maintaining high recall capabilities. This substantial enhancement demonstrates that the minority class samples dynamically generated by ADASYN effectively reshape the data distribution boundaries. Consequently, this enhances the FFNN’s learning and generalization capabilities regarding the deep feature space of pitting rate grades, enabling it to exhibit outstanding classification stability and balance.

#### 3.3.3. Comparison of Confusion Matrices

**Figure 11:**
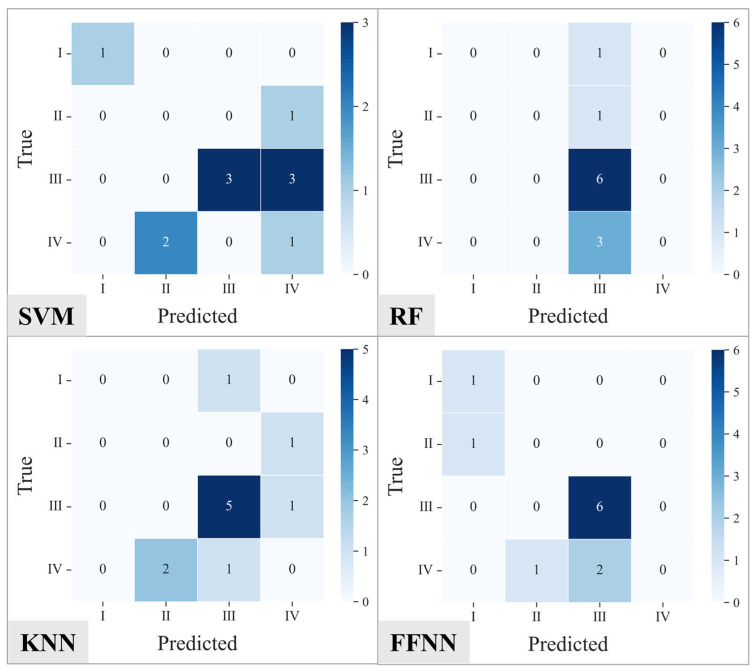
Confusion matrices of the testing results for SVM, RF, KNN, and FFNN models trained on the original dataset.

**Figure 12:**
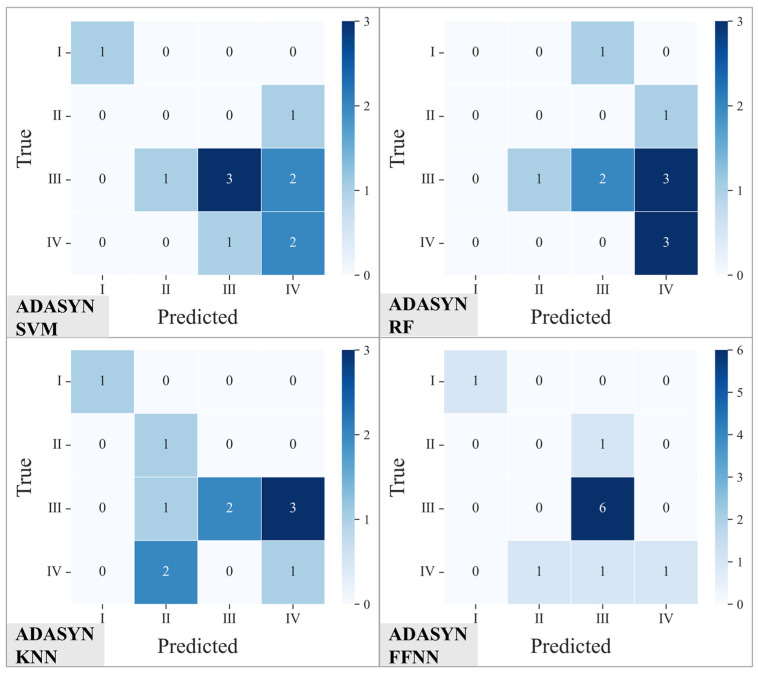
Confusion matrices of the testing results for SVM, RF, KNN, and FFNN models trained on the oversampled dataset.

To further analyze the recognition capabilities of the models for different classes, Figure 11 and Figure 12 present the confusion matrices of their testing results. Figure 11 illustrates the confusion matrix results for SVM, RF, KNN, and FFNN under the condition without oversampling. Overall, all the models exhibit varying degrees of misclassification among different pitting rate grades, and the prediction results show clear characteristics of uneven class distribution. For the SVM model, Grade I samples can be correctly identified, but all Grade II samples are misclassified as Grade IV, and significant confusion occurs between Grades III and IV. This indicates that the model struggles to effectively distinguish adjacent pitting grades given the original imbalanced data. The prediction results of the RF model are highly concentrated in Grade III; most Grade I, II, and IV samples are classified as Grade III, leading to a noticeable increase in non-zero elements outside the main diagonal of the confusion matrix. This suggests a significant class bias phenomenon within the model. The KNN model similarly displays a tendency to concentrate predictions in Grade III, with some Grade IV samples misclassified as Grade II or III, revealing its insufficient discriminative ability for minority classes. In comparison, the FFNN model identifies Grade III samples relatively accurately under the original data, with elements more concentrated along the main diagonal. However, some confusion remains between Grades I/II and Grade III, and a portion of Grade IV samples are misclassified as Grade III, indicating that the class imbalance issue still impacts the model’s performance.

Figure 12 presents the corresponding confusion matrix results for each classification model following ADASYN oversampling treatment. Compared to the original data, the prediction distributions of the various models have changed noticeably, although the extent of improvement varies. For ADASYN-SVM, the distribution of Grade III and IV samples in the prediction results is more dispersed, and some Grade II samples begin to be correctly identified. This illustrates that oversampling alleviates the impact of class imbalance on model discrimination to some extent, yet confusion between adjacent grades persists. The prediction results for ADASYN-RF are primarily distributed in Grades III and IV, while Grade I and II samples remain ineffectively distinguished. The discontinuous distribution along the main diagonal of the confusion matrix indicates limited improvement in classification stability after introducing synthetic samples. ADASYN-KNN shows improvement in recognizing Grade I and II samples, with an increase in elements on the main diagonal. Nevertheless, significant misclassification still occurs for Grade III samples, some of which are classified as Grade IV, resulting in an overall prediction that remains insufficiently balanced. Finally, the confusion matrix of ADASYN-FFNN exhibits the clearest distribution along the main diagonal. All Grade III samples are correctly classified, and the number of misclassifications for Grade I, II, and IV samples is markedly reduced. This demonstrates that the model’s ability to distinguish between different pitting rate grades is significantly enhanced following the oversampling treatment.

The core of the pitting corrosion predictive evaluation lies in accurately defining the corrosion grades to support service state monitoring and risk management. Given the significant asymmetry in the engineering consequences of misclassifying different grades—especially where the underestimation of severe pitting directly threatens equipment safety—model evaluation should transcend the singular pursuit of overall accuracy and focus heavily on the recognition reliability of high-risk grades. Under these requirements, the ADASYN-FFNN model demonstrates discriminative advantages that are highly aligned with engineering practicalities: Not only does it significantly sharpen the decision boundaries between mild pitting rate grades I and II while maintaining a high recognition rate for Grade III pitting, thereby securing an early warning margin for early-stage condition monitoring; but it also successfully achieves the partial correct recall of high-risk Grade IV samples, drastically reducing the false-negative risk of missing severe corrosion.

In summary, integrating the robust FFNN with ADASYN oversampling effectively addresses the challenges of limited and imbalanced datasets in pitting rate classification. This proposed method not only achieves superior performance metrics but aligns well with established corrosion mechanisms and engineering experience, as well. By mitigating data-induced bias, this reliable approach provides practical support for pitting risk evaluation and protection decision-making for stainless steel under complex service conditions.

## 4. Conclusions

In this study, immersion experiments on 316L stainless steel in NaCl and NaClO_3_ solutions were conducted to obtain pitting rate classification data. Based on the results, an ADASYN-FFNN classification model was developed and further compared with three other machine learning models. The classification prediction of the pitting corrosion rates for 316L stainless steel in sodium chloride and sodium chlorate solutions is proposed. The main conclusions are summarized as follows:

(1) The evolution of pitting depth of 316L steel in NaCl and NaClO_3_ solutions is complicated. Based on immersion tests performed at four different temperatures and four different NaClO_3_ concentrations, the maximum pitting depth of 316L stainless steel initially decreases after adding 6 g/L NaClO_3_ as the temperature is below 120 °C for 7 days, and then increases with increasing NaClO_3_ concentration. While, the maximum pitting depth decreases at immersion temperature of 200 °C due to the occurrence of extensive uniform corrosion.

(2) An ADASYN-FFNN-based pitting rate classification model was successfully constructed, realizing the prediction of pitting rate grades for 316L stainless steel in NaCl and NaClO_3_ solutions.

(3) By introducing the ADASYN method into corrosion prediction, the class imbalance problem is effectively alleviated, establishing a balanced data foundation for model training. Consequently, the proposed ADASYN-FFNN model outperforms RF, SVM, and KNN across key metrics such as accuracy, precision, and recall, demonstrating its suitability for the categorical prediction of pitting rate grades.

(4) The proposed pitting rate classification method can provide a valuable idea for corrosion damage assessment in complicated corrosive environment.

## Figures and Tables

**Table 1:** Chemical composition of 316L stainless steel [8].

Element	C	Cr	Ni	Mo	Si	S	P	Mn	Fe
**Content (wt.%)**	0.03	16.24	11.05	2.01	0.72	0.012	0.035	1.93	Bal.

**Table 2:** Classification standards for pitting rates [34].

SY/T 0087.1	Low	Moderate	High	Severe
**Max. pitting rate (mm/a)**	<0.305	0.305–0.611	0.611–2.438	>2.438
**Grade code**	I	II	III	IV

**Table 3:** Statistical results of selected immersion corrosion experiments.

Temperature (°C)	NaClO_3_ (g/L)	pH	Immersion Time (day)	Grade Code
90	0	7	7	I
120	0	2	7	III
…	…	…	…	…
120	0	2	14	II
120	6	2	21	III
120	20	2	30	III
150	40	2	7	IV
150	0	2	7	IV
200	0	7	7	I
200	0	7	14	I
200	0	7	30	III

**Table 4:** Model hyperparameters and search ranges.

Model	Hyperparameter	Search Range [18,49,50,51]
FFNN	hidden1 layer size:	5–20
	hidden2 layer size:	5–20
RandForest	n_estimators:	50–300
	max_depth:	2–30
	min_samples_leaf:	2–20
	min_samples_split:	1–10
SVM	C:	10^−2^–10^3^
	γ:	10^−4^–10^1^
KNN	K:	1–30
	weights:	uniform, distance
	metric:	euclidean, manhattan, minkowski

**Table 5:** Optimal hyperparameters of the models.

Model	Hyperparameter	Optimal Value
FFNN	hidden1 layer size:	9
	hidden2 layer size:	12
RandForest	n_estimators:	221
	max_depth:	25
	min_samples_leaf:	8
	min_samples_split:	5
SVM	C:	92.705
	γ:	0.876
KNN	K:	16
	weights:	uniform
	metric:	manhattan

## Data Availability

The original contributions presented in this study are included in the article. Further inquiries can be directed to the corresponding author.

## Abbreviations

The following abbreviations are used in this manuscript:

ML	Machine Learning
ADASYN	Adaptive Synthetic Sampling
FFNN	Feedforward Neural Network
SVM	Support Vector Machine
ANN	Artificial Neural Network
SMOTE	Synthetic Minority Over-sampling Technique
RF	Random Forest
WOA	Whale Optimization Algorithm
KNN	K-Nearest Neighbors
SEM	Scanning Electron Microscope
OM	Optical Microscope
RMSProp	Root Mean Square Propagation
ReLU	Rectified Linear Unit
SGD	Stochastic Gradient Descent
RBF	Radial Basis Function
TPE	Tree-structured Parzen Estimator
ACC	Accuracy
PRE	Precision
REC	Recall
TP	True Positive
FP	False Positive
FN	False Negative

## References

[1] Costa R.D.F.S., Barbosa M.L.S., Silva F.J.G., Sousa S.R., Sousa V.F.C., Ferreira B.O. (2023). Study of the Chlorine Influence on the Corrosion of Three Steels to Be Used in Water Treatment Municipal Facilities. Materials.

[2] Xie J.J., Ningyu H., Sun X., Yang Zhan J. (2020). Corrosion Behavior of 316L Stainless Steel under Cl− Corrosion Medium. IOP Conf. Ser. Mater. Sci. Eng..

[3] Malik A.U., Mayan Kutty P.C., Siddiqi N.A., Andijani I.N., Ahmed S. (1992). The Influence of pH and Chloride Concentration on the Corrosion Behaviour of AISI 316L Steel in Aqueous Solutions. Corros. Sci..

[4] Akpanyung K.V., Loto R.T. (2019). Pitting Corrosion Evaluation: A Review. J. Phys. Conf. Ser..

[5] Liu G., Tong H., Li Y., Tan Q., Zhu Y. (2021). Passivation Behavior of S136H Steel in Neutral Electrolytes Composed of NaClO_3_ and NaNO_3_ and Its Influence on Micro Electrochemical Machining Performance. Mater. Today Commun..

[6] Safizadeh F., Sorour N., Ghali E., Houlachi G. (2018). Corrosion Behavior of Fe-Mo and Fe-Mo-P Cathodic Coatings in the Simulated Electrolyte for Sodium Chlorate Production. Electrochim. Acta.

[7] Liu G., Shi J., Yang Y., Gong Z., Li C. (2024). Effects of Passivation Behavior on Micro-Electrochemical Machining (ECM) Performance of Stainless Steels with Different Metallographic Phases in NaNO_3_ and NaClO_3_ Solutions. Int. J. Adv. Manuf. Technol..

[8] Yao J., Zhang S., Chen S., He M., Han J., Zhang Z., Chen X. (2026). Competitive Adsorption and Unexpected Corrosion Inhibition Effect of 316L Stainless Steel in NaClO_3_ and NaCl Environment of Chlor-Alkali Industry. Corros. Sci..

[9] Dobosz L.M. (2012). Electrochemical Assessment of Corrosion Behavior of 316 Stainless Steel in Sodium Chlorate Solutions. NACE Int. Corros. Conf. Ser..

[10] (2018). Standard Test Method for Conducting Cyclic Potentiodynamic Polarization Measurements for Localized Corrosion Susceptibility of Iron-, Nickel-, or Cobalt-Based Alloys.

[11] (2018). Standard Test Method for Electrochemical Critical Pitting Temperature Testing of Stainless Steels and Related Alloys.

[12] (2015). Standard Test Methods for Pitting and Crevice Corrosion Resistance of Stainless Steels and Related Alloys by Use of Ferric Chloride Solution.

[13] (2021). Standard Guide for Examination and Evaluation of Pitting Corrosion.

[14] Jiménez-Come M.J., Turias Domínguez I.J., Matres V. (2017). Prediction of Pitting Corrosion Status of EN 1.4404 Stainless Steel by Using a 2-Stage Procedure Based on Support Vector Machines. J. Chemom..

[15] Jiménez-Come M.J., Turias I.J., Ruiz-Aguilar J.J. (2015). Pitting Corrosion Behaviour Modelling of Stainless Steel with Support Vector Machines. Mater. Corros..

[16] Jiménez-Come M.J., de la Luz Martín M., Matres V. (2019). A Support Vector Machine-Based Ensemble Algorithm for Pitting Corrosion Modeling of EN 1.4404 Stainless Steel in Sodium Chloride Solutions. Mater. Corros..

[17] Jafari E., Jafari A., Hadianfard M.J. (2011). Prediction of Pitting Corrosion of Surface Treated AISI 316L Stainless Steel by Artificial Neural Network. Corros. Eng. Sci. Technol..

[18] Aghaaminiha M., Mehrani R., Colahan M., Brown B., Singer M., Nesic S., Vargas S.M., Sharma S. (2021). Machine Learning Modeling of Time-Dependent Corrosion Rates of Carbon Steel in Presence of Corrosion Inhibitors. Corros. Sci..

[19] Jiménez–Come M.J., Turias I.J., Trujillo F.J. (2014). An Automatic Pitting Corrosion Detection Approach for 316L Stainless Steel. Mater. Des. (1980-2015).

[20] Jalili S., Jaberi A., Mahjani M.G., Jafarian M. (2008). Prediction of Lead Corrosion Behavior Using Feed-Forward Artificial Neural Network. J. Iran. Chem. Soc..

[21] Hu Q., Liu G., Li B., Hu S., Chen J., Liu H., Agaian S.S. (2025). Study on Prediction of Soil Corrosion in Power Grounding Based on Artificial Neural Network. Proceedings of the Second International Conference on Big Data, Computational Intelligence, and Applications (BDCIA 2024), Huanggang, China, 15–17 November 2024.

[22] Pascal M. (2026). Hybrid Feedforward Neural Network for Pressure Vessel Internal Corrosion Prediction: Integrating Chemical Models with Inspection Data for Structural Integrity Assessment. Fract. Struct. Integr..

[23] Gosain A., Sardana S. Handling Class Imbalance Problem Using Oversampling Techniques: A Review. Proceedings of the 2017 International Conference on Advances in Computing, Communications and Informatics (ICACCI).

[24] Jiang K., Lu J., Xia K. (2016). A Novel Algorithm for Imbalance Data Classification Based on Genetic Algorithm Improved SMOTE. Arab. J. Sci. Eng..

[25] Swana E.F., Doorsamy W., Bokoro P. (2022). Tomek Link and SMOTE Approaches for Machine Fault Classification with an Imbalanced Dataset. Sensors.

[26] Sutojo T., Rustad S., Akrom M., Syukur A., Shidik G.F., Dipojono H.K. (2023). A Machine Learning Approach for Corrosion Small Datasets. npj Mater. Degrad..

[27] Terrados-Cristos M., Diaz-Piloneta M., Ortega-Fernández F., Martinez-Huerta G.M., Alvarez-Cabal J.V. (2025). Corrosion Risk Assessment in Coastal Environments Using Machine Learning-Based Predictive Models. Sensors.

[28] Wan H., Liu S., Song D., Cai W., Wang Y., Geng L., Chen C. (2025). Prediction of Industrial Atmospheric Corrosion and Evaluation of Influencing Factors Based on Machine Learning. J. Mater. Eng. Perform..

[29] Patel P., Aryai V., Arzaghi E., Kafian H., Abbassi R., Garaniya V. (2025). Classification of Pitting Corrosion Damage in Process Facilities Using Supervised Machine Learning. Can. J. Chem. Eng..

[30] He H., Bai Y., Garcia E.A., Li S. ADASYN: Adaptive Synthetic Sampling Approach for Imbalanced Learning. Proceedings of the 2008 IEEE International Joint Conference on Neural Networks (IEEE World Congress on Computational Intelligence).

[31] Ahmed G., Er M.J., Fareed M.M.S., Zikria S., Mahmood S., He J., Asad M., Jilani S.F., Aslam M. (2022). DAD-Net: Classification of Alzheimer’s Disease Using ADASYN Oversampling Technique and Optimized Neural Network. Molecules.

[32] Saqib S.M., Mazhar T., Iqbal M., Shahazad T., Almogren A., Rehman A.U., Hamam H. (2025). Enhancing Electricity Theft Detection with ADASYN-Enhanced Machine Learning Models. Electr. Eng..

[33] Zhang Y., Qiao J., Li T., Ma C., Zeng P., Li S., Zou M., Zheng X. (2026). An Improved LightGBM Model with ADASYN and Whale Optimization Algorithm for Rockburst Intelligent Prediction. Rock Mech. Rock Eng..

[34] (2018). Standard for Corrosion Evaluation of Steel Pipelines and Storage Tanks Part 1: Direct Evaluation of External Corrosion of Buried Steel Pipelines.

[35] Altabey W.A. (2016). FE and ANN Model of ECS to Simulate the Pipelines Suffer from Internal Corrosion. Struct. Monit. Maint..

[36] Kubisztal J., Kubisztal M., Haneczok G. (2020). Corrosion Damage of 316L Steel Surface Examined Using Statistical Methods and Artificial Neural Network. Mater. Corros..

[37] Lo M., Vijaya Kumar S.D., Karuppanan S., Ovinis M. (2022). An Artificial Neural Network-Based Equation for Predicting the Remaining Strength of Mid-to-High Strength Pipelines with a Single Corrosion Defect. Appl. Sci..

[38] Ossai C.I. (2019). A Data-Driven Machine Learning Approach for Corrosion Risk Assessment—A Comparative Study. Big Data Cogn. Comput..

[39] Hornik K., Stinchcombe M., White H. (1989). Multilayer Feedforward Networks Are Universal Approximators. Neural Netw..

[40] Cybenko G. (1989). Approximation by Superpositions of a Sigmoidal Function. Math. Control Signals Syst..

[41] Hornik K. (1991). Approximation Capabilities of Multilayer Feedforward Networks. Neural Netw..

[42] Romanowicz P.J., Szybiński B., Barski M., Stawiarski A., Pałac M. (2025). Application of FEM Analyses and Neural Networks Approach in Multi-Stage Optimisation of Notched Steel Structures Subjected to Fatigue Loadings. Appl. Sci..

[43] Santurkar S., Tsipras D., Ilyas A., Madry A. (2018). How Does Batch Normalization Help Optimization?. Proceedings of the Advances in Neural Information Processing Systems, Montreal, QC, Canada, 3–8 December 2018.

[44] Srivastava N., Hinton G., Krizhevsky A., Sutskever I., Salakhutdinov R. (2014). Dropout: A Simple Way to Prevent Neural Networks from Overfitting. J. Mach. Learn. Res..

[45] Bishop C.M. (2006). Pattern Recognition and Machine Learning.

[46] Kingma D.P., Ba J. (2017). Adam: A Method for Stochastic Optimization. arXiv.

[47] Claesen M., Moor B.D. (2015). Hyperparameter Search in Machine Learning. arXiv.

[48] Akiba T., Sano S., Yanase T., Ohta T., Koyama M. (2019). Optuna: A next-Generation Hyperparameter Optimization Framework. Proceedings of the 25th ACM SIGKDD International Conference on Knowledge Discovery & Data Mining, Anchorage, AK, USA, 4–8 August 2019.

[49] Kheradmanda S., Nematollahi O., Ayoobia A.R. (2016). Clearness Index Predicting Using an Integrated Artificial Neural Network (ANN) Approach. Renew. Sustain. Energy Rev..

[50] Jarren L.C., Gazenbiller E., Arya V., Reitz R., Oechsner M., Feiler C., Zheludkevich M.L., Höche D. (2025). Machine Learning–Assisted Risk Assessment of Pitting Corrosion Susceptibility of AA1050 in Ethanol-Containing Fuels. Mater. Corros..

[51] Al-Zamzami M., Al-Gheethi A., Alzaeemi S.A., Al-Sahari M., Al-Maqtari Q., Noman E. (2024). Validity of Zinc Oxide Nanoparticles Biosynthesized in Food Wastes Extract in Treating Real Samples of Printing Ink Wastewater; Prediction Models Using Feed-Forward Neural Network (FFNN). Chemosphere.

